# Bovine fasciolosis at increasing altitudes: Parasitological and malacological sampling on the slopes of Mount Elgon, Uganda

**DOI:** 10.1186/1756-3305-5-196

**Published:** 2012-09-07

**Authors:** Alison Howell, Lawrence Mugisha, Juliet Davies, E James LaCourse, Jennifer Claridge, Diana JL Williams, Louise Kelly-Hope, Martha Betson, Narcis B Kabatereine, J Russell Stothard

**Affiliations:** 1 Disease Control Strategy Group, Liverpool School of Tropical Medicine, Pembroke Place, Liverpool, L3 5QA, United Kingdom; 2 College of Veterinary Medicine, Animal Resources & Biosecurity, Makerere University, Kampala, Uganda; 3 Conservation & Ecosystem Health Alliance (CEHA), P.O. Box 34153, Kampala, Uganda; 4 Veterinary Parasitology, Institute of Infection and Global Health, School of Veterinary Science, University of Liverpool, Liverpool, L69 7ZJ, United Kingdom; 5 Vector Control Division, Ministry of Health, Kampala, Uganda

## Abstract

**Background:**

To clarify the extent and putative transmission zone of bovine fasciolosis on the slopes of Mount Elgon, Uganda, conjoint parasitological and malacological surveys, inclusive of inspection of animals at slaughter, were undertaken at increasing altitudes.

**Results:**

A total of 239 cattle were sampled across eight locations ranging in elevation from 1112-2072 m. Faecal material was examined for presence of *Fasciola* eggs and sera were tested by ELISA for antibodies against *Fasciola* antigens. Bolstering this, 38 cattle at slaughter from 2 abattoir sites at 1150 m and 1947 m were inspected; in addition, wild buffalo stool (n = 10) opportunistically picked within Mount Elgon National Park (MENP) at 3640 m was examined. By faecal egg detection, prevalence of *Fasciola gigantica* at low (<1500 m) and high (>1500 m) altitude sites was 43.7% (95% CI 35.4-52.2) and 1.1% (95% CI 0.0-6.0), respectively, while by ELISA was much higher, low altitude - 77.9% (95% CI 69.7-85.4) and high altitude - 64.5% (95% CI 51.3-76.3). The decline in prevalence with increasing altitude was corroborated by abattoir sampling. Thirty seven aquatic habitats, ranging from 1139-3937 m in altitude were inspected for freshwater snails, 12 of which were within MENP. At lower altitudes, *Lymnaea (Radix) natalensis* was common, and often abundant, but at higher altitudes became much rarer ceasing to be found above 1800 m. On the other hand, *Lymnaea (Galba) truncatula* was found only at altitudes above 3000 m and within MENP alone. The snail identifications were confirmed by DNA analysis of the ribosomal 18S gene.

**Conclusions:**

Active infections of *F. gigantica* in cattle are common in lower altitude settings but appear to diminish with increasing elevation. This is likely due to a growing paucity of intermediate hosts, specifically populations of *L. natalensis* for which a natural boundary of 1800 m appeared. Although *F. hepatica* was not encountered, the presence of several populations of *L. truncatula* at elevations over 3000 m point towards a potential transmission zone within MENP should this parasite be introduced.

## Background

Fasciolosis, caused by infection with the liver fluke *Fasciola*, can cause significant economic losses in African livestock [1,2]. The complex nature of the lifecycle and epidemiology of this snail-borne disease presents challenges for predictive mapping at the herd-level, as well as disease management and animal husbandry at the individual-level [3]. *Fasciola gigantica* and *Fasciola hepatica* can infect a wide variety of domesticated animals, wildlife and people [4-9]. Thus the disease-endemic zone can be difficult to define from parasitological data alone and so consideration of the distribution of associated snail intermediate hosts can be important [10]. *F. gigantica* is the most common liver fluke in sub-Saharan Africa, being adapted to warmer conditions [11] likely due to the widespread distribution of its intermediate host *Lymnaea (Radix) natalensis *[12]. On the other hand owing to a more limited distribution of its intermediate host *Lymnaea (Galba) truncatula *[12], *F. hepatica* can exist in zoonotic foci which are more restricted to cooler regions of Africa, including Kenya, Ethiopia and Tanzania [1,10,13]. Nonetheless, actual or potential overlap of both types of fasciolosis can occur especially where snail-habitats converge, for example, with increasing altitude as in the highlands of Ethiopia [14] or perhaps in upland zones of eastern Uganda, as yet to be fully explored. In the Mount Elgon area of Uganda, fasciolosis is poorly studied as there is no systematic veterinary or medical disease surveillance system.

Cattle are Uganda’s most economically important livestock species with an estimated population of 11 million [15]. The majority are either indigenous Zebu or Sanga, with less than 5% being imported ‘exotic’ breeds, mainly Friesians [16]. Livestock production is hampered by many disease constraints of which fasciolosis is considered the most important helminth infection [17]. The Mount Elgon region consists of predominately rural subsistence farmers covering a zone between 1000-2300 m in altitude rising towards an important wildlife reserve, the Mount Elgon National Park (MENP). Encompassing a total area of some 1,145 km^2^, MENP commences at 2300 m and extends to 4321 m at Wagagi Peak. Within the park, a number of herds of wild ruminants are known including buffalo, antelope and elephant but illegal cattle trading routes, from Uganda to Kenya and *vice versa*, traverse throughout. However, with increasing Uganda Wildlife Authority (UWA) foot patrols servicing an increasing hiking and camping tourism, illegal cattle trafficking has declined in recent years.

Like elsewhere in Uganda, the lowland areas of Mount Elgon are known to be endemic zones for *F. gigantica* with reports documenting the prevalence of *F. gigantica* at 54.7% in cattle [16,18,19]. A contemporary situational analysis, however, is yet lacking. From a malacological perspective, there has been no update to the formal snail surveys conducted by Georg Mandahl-Barth and by Hubendick in their general treatise on Ugandan freshwater snails and *Lymnaea*, respectively over 50 years ago [12,20]. Both *Lymnaea natalensis* and *Lymnaea mweruensis* have been reported from the area with the latter species now considered synonymous with *Lymnaea truncatula*, as collected by C.C. Cridland from Sasa River Camp at 2900 m (now within MENP). Such upland areas, like those in neighbouring Tanzania, are thought suitable zones for the transmission of *F. hepatica*, for example, *L. truncatula* being recently found at 2712 m & 2720 m with identifications confirmed by DNA analysis of the ribosomal 18S [10]. Though *F. hepatica* has yet to be encountered in natural transmission cycles in Uganda, it has been known from earlier reports within UK-imported cattle [21].

Clearly defining such local zones of transmission in eastern Uganda is also important for further modelling of the suitability of habitats elsewhere in East Africa. Various authors have designed models based on climate and intermediate host presence to predict the prevalence of *Fasciola* spp. [14,22]. However, areas that appear broadly similar in terms of climate can have very different snail populations due to variations in micro-climate and local aquatic factors, e.g. water pH and conductivity. This limits the accuracy of such climatic models, and localised parasitological and malacological data are still required for prediction of actual disease zones or outbreaks [22,23]. In many countries, signalment of cattle condition [24] and subsequent meat inspection provides an opportunity to monitor the incidence of fasciolosis, also allowing access to adult worms enabling morphological identification [23,25]. However, it is not able to detect past infections in those animals that have either been treated or developed immunity and self-cured. A suitable immunological test could fill this gap, and also detect pre-patent infections, but presently this is only available for assaying antibody titres in cattle to excretory/secretory (ES) antigens of *F. hepatica *[26]. With this assay, heterologous reactions to *F. gigantica* are likely but as yet not known, however, serological testing should be an interesting adjunct in revealing putative transmission zones.

Using a combination of parasitological sampling, bolstered by experimental serology, our study aimed to investigate the occurrence of fasciolosis in bovids at low and high altitude areas on the slopes of Mount Elgon and also assessed animal condition (i.e. body signalment). The parasitological surveys were complemented with a conjoint malacological appraisal in an attempt to better define the actual or potential disease transmission zone of these parasites.

## Methods

### Study area and design

**Figure 1:**
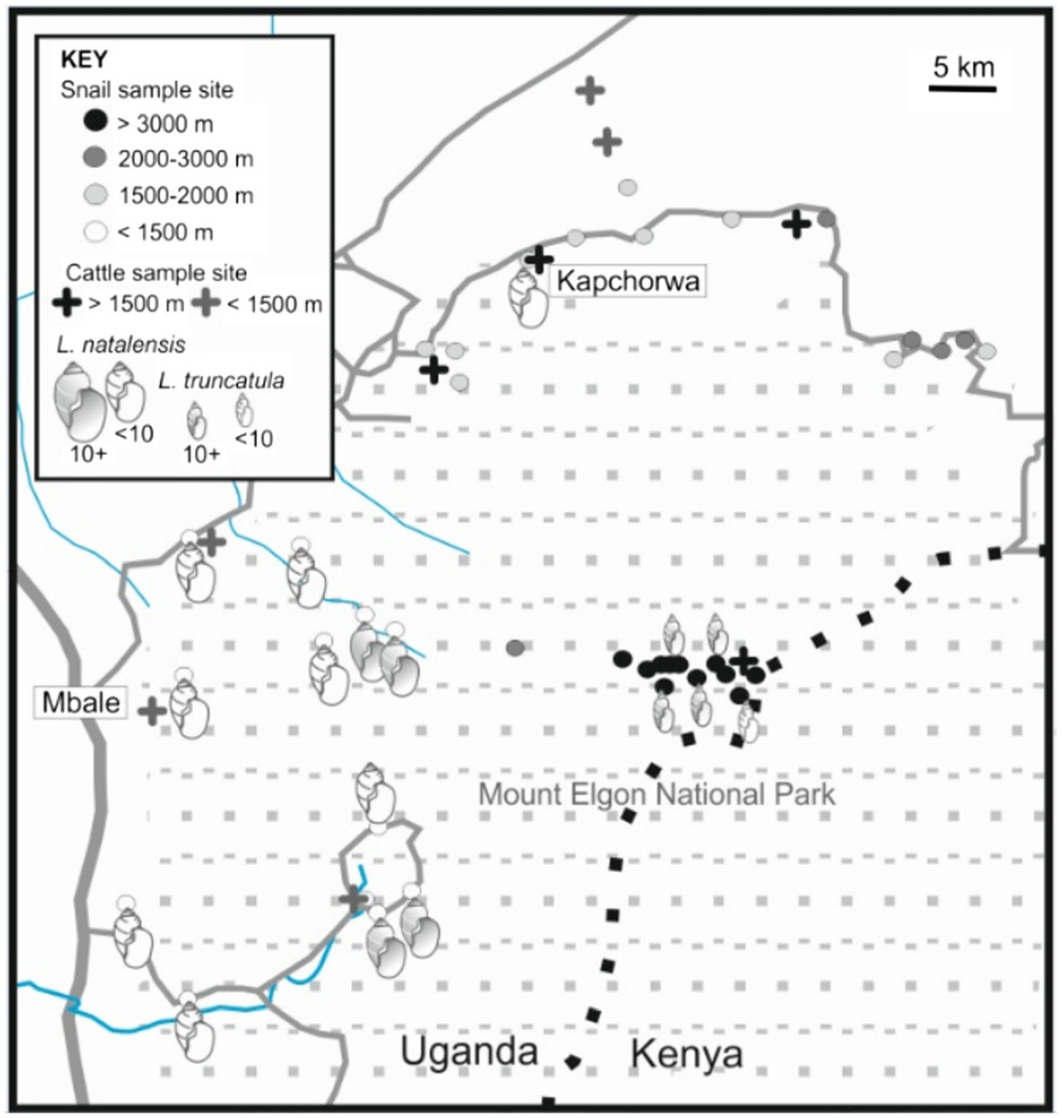
**Lymnaeid snails found at various sites in the Mount Elgon region.** Sites are coded by altitude (background map redrawn from Google, 2011 http://maps.google.co.uk/maps).

A preliminary visit to the Mount Elgon study area took place in March 2011 by the corresponding author when local arrangements were made with the District Administration Offices for future surveys in June-July and entry into MENP. In addition, a selection of 20 freshwater habitats was identified and spot-surveyed for freshwater snails for later comparison within the year. In June-July 2011, a cross-sectional study was conducted; sampling of cattle centred around six main sites of varying altitudes: Mbale (1150 m), Sironko (1155 m), Bududa (1268 m), Sipi (1856 m), Kapchorwa (1947 m) and Kween (2072 m). In addition, cattle were also sampled from larger roving herds in two sites between Kapchorwa (1112 m) and Ngenge (1468 m), Figure 1. Sample size calculations were performed using WinEpiscope 2.0 (University of Edinburgh, UK). For 80% power, 95% confidence, an estimated prevalence of 40% at the low altitude (<1500 m) sites [18,27] an estimated prevalence of 20% at the high altitude (>1500 m) sites (assumed to be lower based on absence of lymnaeids found in a preliminary study in March 2011) a sample size of 80 cattle was required from high and low altitude sites.

At each of the six main sites, at least 30 cattle were sampled, a maximum of two per owner in order to avoid bias due to multiple animals from the same herd having similar risk factors. Where possible, stool and blood specimens were taken from each animal at the time of tethering. At four of these sites, convenience sampling was employed, with local community leaders mobilising farmers to bring their animals to a central point. At two of the sites this method was unsuccessful and individual households around a central point were visited. From the two large roving herds in between Kapchorwa and Ngenge, a random selection of 10 cattle from each were sampled. Additionally, during a three-day trek on foot to altitudes of between 3000-4000 m in MENP, freshly deposited buffalo stool samples (n = 10) were opportunistically picked from the ground at an elevation of 3900 m at a unique grazing site adjacent to a hot spring.

### Signalment of cattle

The data on signalment of each cow were recorded as follows: Age category - calf (approximately 2 weeks to 6 months), sub-adult (6 months to 18 months), or adult (18 months plus); sex; breed - Friesian, local (zebu type) or hybrid; and body condition score (BCS) graded through 1 to 5 classifications according to Roche [24].

### Faecal egg detection

Faecal samples were obtained per rectum or taken from the ground if seen to have been directly produced. Approximately 3 g of stool was thoroughly mixed into 250 ml of bottled mineral water containing 0.5% *Tween*-20 (Sigma-Aldrich, UK) before filtration-concentration; two methods were each used for the isolation of eggs from faeces. ‘*Individual stool’* was analysed using Flukefinder® kit (see http://www.flukefinder.com) and in an attempt to confirm these individual findings ‘*pooled stool of 10 animals’* was subjected to standard coprological filtration with a descending pore series of 3 large-diameter metal sieves (at 425, 125, and 32 microns respectively) following [10]. Faecal eggs, from liver flukes or paramphistomes, were finally collected in a 10 cm glass petri-dish stained with a few drops of 10% methylene blue solution, viewed and counted under the dissecting microscope at x40 magnification [28].

### Blood sampling and serology

Blood was obtained from an ear vein and harvested into sterile 10 ml plastic syringes and allowed to clot in a 1.5 ml eppendorf tube. Serum was then separated by centrifugation, and heat-inactivated by incubation at 56°C for 30 min, as required for importation licensing by the Department for the Environment, Food and Rural Affairs (DEFRA), UK. After heat-inactivation samples were stored in liquid nitrogen before transportation to the UK. ELISA was performed according to [26,29] with the following minor modifications: The concentration of *F. hepatica* E/S antigens used to coat the ELISA plate was 1 mg/ml; the concentration of monoclonal anti-bovine IgG used was 1:70,000 (this was first optimised by a checkerboard titration); and 20 minutes following the addition of TMB substrate, 100 μl of stopping solution (0.5 M HCL) was added to each well prior to reading. The results are given as the mean of the optical density (OD) obtained from duplicate samples expressed as a percentage of the strong positive control (PP), with PP of 15 or above considered a positive result [26,29].

### Liver necropsy

Livers obtained from cattle slaughtered at Mbale (n = 30) and Kapchorwa (n = 8) abattoirs, at 1150 m and 1947 m, were inspected for flukes by cutting open the main bile ducts into the liver parenchyma. Adult flukes were identified morphologically based on size and shape [30,31]. Faecal and blood samples were taken from the large intestine and mesenteric vein respectively and processed as described above for ELISA.

### Malacological sampling

In total 37 freshwater sites, ranging in altitude from 1139 m to 3937 m above sea level, were selected and surveyed for aquatic snails. Sites were chosen to include a variety of streams, marshes and pools to cover as wide an area as possible, within easy reach of vehicular access, with the exception of the sites above 3000 m within MENP that were visited on foot. Using collecting sieves and snail scoops, two people surveyed each site for 10 minutes and all collected snails were counted. If there were different types of habitat within each location, for example, slow/fast flowing water within streams or drainage ditches, these were all surveyed. The presence and numbers of each species of snail were recorded according to field identification keys of Brown [32]. To later confirm the identification of encountered lymnaeids, a selection of snails was placed in 70% ethanol for DNA analysis. Spot-site water chemistry readings were taken for pH, conductivity, total dissolved salt and temperature from each of the different habitats using a handheld water meter (Hanna H1-9816-6; VWR, UK) to investigate ecological associations.

### DNA-based snail identification

Genomic DNA was extracted from a total of 16 snails representative of *L. natalensis* (n = 8) and *L. truncatula* (n = 8) using the DNeasy Blood and Tissue Kit (QIAGEN, Germany). A 450 base pair region of the nuclear ribosomal 18S was amplified by PCR with the primers 18SLYMFOR 5′ agtagtcatatgcttgtctcaaagattaagcca and 18SLYMREV, 5′ tgcgcgcctctgccttccttggatgtggtagccgt, following Stothard [33]. Amplification products were purified using the QIAquick PCR Purification Kit (QIAGEN, Germany) and sequenced using the ABI PRISM™ BigDye Terminator Cycle Sequencing Ready Reaction Kit (Applied Biosystems, UK). Sequencing chromatograms were produced by the DNA Sequencing Facility, The Natural History Museum, London, UK and analyzed using DNASTAR’s Lasergene Sequence Analysis Software (Madison, USA). Compiled sequences were aligned and also compared with other lymnaeids on GenBank. The putative secondary structure of the variable V2 E10-1 helix was investigated using the RNAfold web server (http://rna.tbi.univie.ac.at/) and compared to that described by Stothard and Bargues & Mas-Coma [33,34]. To investigate known restriction site variation, amplification products were also digested, separately, with either *msp*I or *cfo*I enzymes and subsequently separated by PAGE and ethidium bromide staining according to [33], and photographed with a Gel Doc E2 Imager (BioRad, UK).

### Statistical analysis

For raw prevalence data, exact binomial confidence intervals were calculated for the cattle surveys using Stata v. 9 (2007, Texas, 77845). Other tests were performed using PASW v. 18 (2010, SPSS Inc, Chicago). Poisson regression was used for multivariate association with snail data and recorded environmental variables. Firstly, parameters with close correlations (>0.8) were excluded from being modelled together. Secondly, each parameter was entered separately and the model with the smallest difference between scaled deviance and degrees of freedom taken as being the most representative.

## Results

The signalment data (i.e. body condition) recorded for each inspected animal is shown in Table 1. Many of the cattle were often subjected to zero grazing, especially in peri-rural settlements, whereby animal holders provided on-site food to tethered animals.

**Table 1:** Biometric data assessing signalment of cattle sampled for fluke infection at high (n = 145) and low (n = 94) altitudes [95% confidence intervals are shown]

	**Low altitude < 1500 m**	**High altitude > 1500 m**
	**Percentage (95%CI)**	**Percentage (95 % CI)**
Breed		
Local	39.3 (31.3-47.8)	23.4 (15.3-33.3)
Hybrid	20.0 (13.8-27.4)	34.0 (24.6-44.5)
Friesian	19.3 (13.2-26.7)	38.3 (28.5-48.9)
Unknown		
Age		
Calf	14.5 (9.2-21.3)	14.9 (8.4-23.7)
Sub-adult	11.7 (7.0-18.1)	28.7 (19.9-39.0)
Adult	73.8 (65.9-80.7)	56.4 (45.8-66.6)
Body condition score		
1	0.9 (0.0-4.8)	4.4 (1.2-10.9)
2	15.9 (9.7-24.0)	31.9 (22.5-42.5)
3	65.5 (56.0-74.2)	55.0 (44.2-65.4)
4	17.7 (11.2-26.0)	7.7 (3.2-15.2)
5	0.0 (0.0-3.2)	1.1 (0.0-6.0)
Underweight (BCS 1-2)	16.8 (10.4-25.0)	36.3 (26.4-47.0)
Sex		
Male	27.4 (19.5-36.6)	16.3 (9.2-25.8)
Female	72.6 (63.4-80.5)	83.7 (74.2-90.8)

### Faecal egg count analysis

Faecal samples obtained from 233 cattle were processed individually with testing on-site near the point of collection. From the high altitude population, only one cow was found to be egg positive for *Fasciola*, with only a single egg observed in this animal’s faeces. In the low altitude population, 43.7% (95% CI 35-52) of cattle tested were egg positive for *Fasciola* by Flukefinder®, Table 2. Faecal egg counts ranged from 1 to 43 eggs per gram (epg) of stool, with the majority being low numbers of eggs (<10). The results from the pooled stool samples were in all cases qualitatively the same as the Flukefinder® results with the exception that eggs were not found at altitudes above 1500 m. All of the buffalo samples were found negative for eggs of *Fasciola.* Eggs of paramphistomes were often seen with prevalence estimated by Flukefinder® to be 74% (95% CI = 66.4-81.2%) and 58% (95% CI = 47.8-69.1) at low and high altitude sites, respectively. Faecal egg counts for amphistomiasis ranged from 1 to over 250, with low altitude cattle showing a higher intensity of excreted eggs. Of the buffalo faecal samples, no egg of *Fasciola* was seen but 6 out of 10 were positive for amphistome eggs. There were no significant relationships between liver fluke infection and either BCS, breed, sex, or paramphistome infection.

**Table 2:** **Prevalence of *Fasciola *infections as detected by faecal egg detection, ELISA and gross liver inspection at abattoir [95% confidence intervals are shown in brackets]**

**Test**	**Low altitude < 1500 m**		**High altitude > 1500 m**	
	**Prevalence**	**n**	**Prevalence**	**n**
Faecal exam	43.7 (35.4-52.2)	142	1.1 (0.0-6.0)	91
Gross liver	48.4 (30.2-66.9)	30	0 (0-37.9)	8
ELISA	77.9 (69.7-85.4)	104	64.5 (51.3-76.3)	62

### Serological analysis

Owing to resource constraints and sampling spoiling, a sub-set of animals were tested by ELISA, finding that 64.5% (95% CI = 51.3-76.3) of cattle at high altitude were positive for *Fasciola* spp. whereas 77.9% (95% CI = 69.7-85.4) of cattle at low altitude were positive, Table 2. This was a statistically significant difference, however, as the high ELISA positive rate was not confirmed by egg detection in the high altitude samples (i.e. confirmation of active infections), only egg detection results are used for subsequent analysis and further discussion.

### Liver necropsy

Livers from 30 animals originating at low altitude and 8 from high altitude were inspected. The proportion of animals infected with liver fluke at the low altitude sites was 48.4% (95% CI 30.2-66.9). At the high altitude sites, none (95% CI 0.0-37.9) of the animals were found to be infected, Table 2. Owing to time constraints within slaughter houses, numbers of flukes found were not recorded, but in the majority of animals less than 10 flukes were found. Based on body size and shape, all flukes found were confidently identified as *F. gigantica*, there was no evidence of *F. hepatica*-like worms.

### Malacological surveys

*Lymnaea natalensis* was found at 12 sites ranging in altitude from 1139 m to 1770 m. By contrast *L. truncatula* was found at only 5 sites, all above 3500 m. There was no geographical overlap between the two species, with the zone between 1800 m and 3500 m being a lymnaeid-free zone, Figure 1.

**Figure 2:**
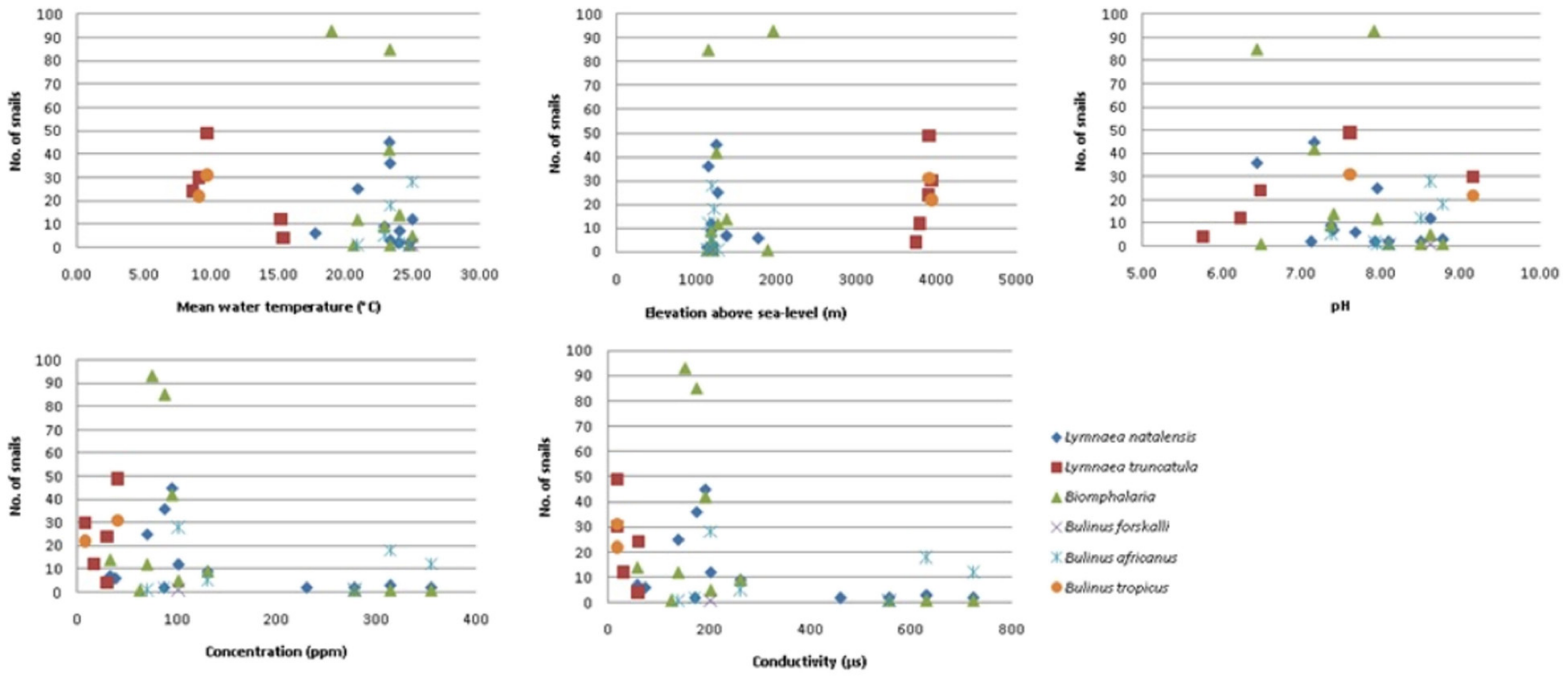
**Correlations between number of snails found at a site, and the mean temperature, pH, conductivity and concentration of the water and elevation.** The strongest correlations are for temperature and altitude.

Correlation analysis showed significant correlation of snail numbers with altitude and water temperature for both species. Dissolved salt concentration and electrical conductivity showed significant correlation with *L. truncatula* numbers only, Figure 2.

Using Poisson regression, altitude, pH and conductivity appeared to be predictors of *L. natalensis* and *L. truncatula* with contrasting associations, Table 3. The location and numbers of snails collected alongside the presence of other aquatic gastropod genera is shown in Table 4.

**Table 3:** Results of Poisson regression on collected snail and aquatic habitat data

	**Included in model**	**Relative risk**	**p**	**Scaled deviance:df**
*L. natalensis*	Elevation (100 m)	0.58	<0.01	3.18
	pH	0.65	0.07	
	Concentration (10 ppm)	0.90	<0.01	
*L. truncatula*	Elevation (100 m)	10.97	<0.01	1.10
	pH	0.33	<0.01	
	Conductivity (10 μs)	0.58	<0.01	

**Table 4:** Location and occurrence of snails at each of the 37 collection sites

**Site no.**	**GPS location (in decimal seconds)**	**Altitude (m)**	**Habitat**	***Lymnaea*spp. (n)**	**Other genera present**
1	N01°13.908 E034°17.418	1139	Stream	*L. natalensis* (2)	*Biomphalaria, Bulinus*
2	N01°00.128 E034°21.545	1150	Pond	*L. natalensis* (36)	*Biomphalaria*
3	N01°13.131 E034°13.899	1152	Stream	*L. natalensis* (2)	*Biomphalaria, Bulinus*
4	N00°59.982 E034°11.602	1167	Ditch	*L. natalensis* (2)	-
5	N00°56.932 E034°14.947	1184	Ditch	*L. natalensis* (9)	*Biomphalaria, Bulinus*
6	N01°07.714 E034°13.135	1187	Stream	*L. natalensis* (2)	*Bulinus*
7	N01°11.157 E034°19.372	1188	River	*L. natalensis* (12)	*Biomphalaria, Bulinus*
8	N01°09.607 E034°17.994	1215	Stream	*L. natalensis* (3)	*Biomphalaria, Bulinus*
9	N01°09.833 E034°20.897	1251	Stream	*L. natalensis* (45)	*Biomphalaria*
10	N01°00.680 E034°20.111	1268	Stream	*L. natalensis* (25)	*Biomphalaria, Bulinus*
11	N01°03.040 E034°20.662	1375	Stream	*L. natalensis* (7)	*Biomphalaria*
12	N01°20.288 E034°22.771	1645	Waterfall	-	-
13	N01°26.102 E034°29.626	1718	Stream	-	-
14	N01°21.224 E034°23.318	1770	Stream	*L. natalensis* (6)	-
15	N01°23.037 E034°25.247	1810	Stream	-	-
16	N01°24.248 E034°29.843	1883	Stream	-	*Biomphalaria*
17	N01°20.030 E034°23.364	1896	Stream	-	-
18	N01°23.653 E034°26.894	1922	Stream	-	-
19	N01°24.854 E034°32.485	1951	Stream	-	*Biomphalaria*
20	N01°20.182 E034°39.728	1980	Stream	-	-
21	N01°20.337 E034°42.581	1998	Stream	-	-
22	N01°20.469 E034°41.848	2021	Stream	-	-
23	N01°20.311 E034°40.370	2021	Stream	-	-
24	N01°24.864 E034°37.238	2036	Stream	-	-
25	N01°20.087 E034°41.370	2069	Stream	-	-
26	N01°10.471 E034°26.434	2900	Stream	-	-
27	N01°09.757 E034°29.491	3573	Stream	-	-
28	N01°08.746 E034°34.199	3640	Stream	-	-
29	N01°08.716 E034°33.667	3746	Bog	*L. truncatula* (4)	-
30	N01°09.067 E034°32.644	3779	Stream	-	-
31	N01°08.972 E034°32.842	3788	Bog	*L. truncatula* (12)	-
32	N01°09.463 E034°30.478	3812	Bog	-	-
33	N01°09.306 E034°31.170	3829	Stream	-	-
34	N01°09.348 E034°30.757	3870	Bog	-	-
35	N01°09.324 E034°31.401	3902	Bog	*L. truncatula* (24)	-
36	N01°09.305 E034°31.536	3905	Pool/bog	*L. truncatula* (49)	*Bulinus*
37	N01°08.944 E034°30.634	3937	Pool	*L. truncatula* (30)	*Bulinus*

### DNA-based snail identification

**Figure 3:**
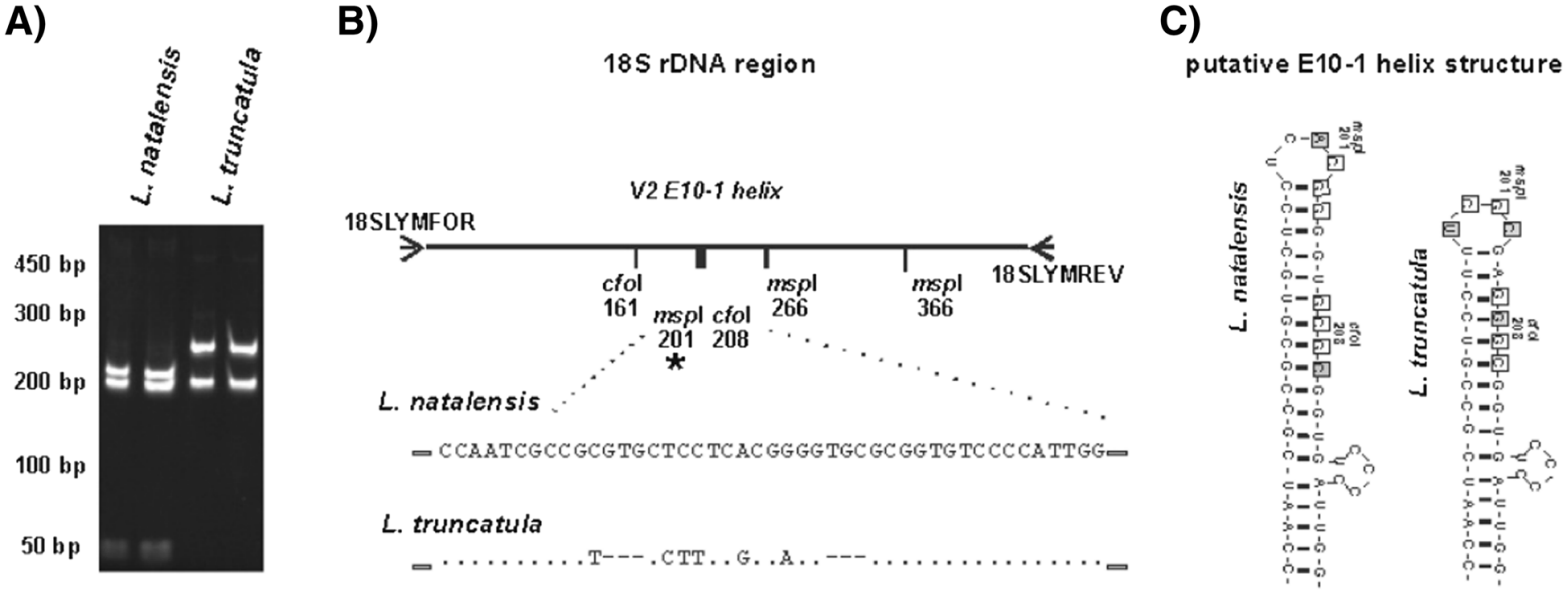
**Molecular characterization of *Lymnaea. *A**) RFLP analysis of 18S region with *cfo*I. **B**) Schematic RFLP map of the 18S region with corresponding DNA sequence variation within the E10-1 helix. **C**) Putative secondary structure of the E10-1 helix illustrating the RFLP variation and nucleotide differences.

RFLP examination and DNA sequence analysis of the 450 bp PCR fragment confirmed the presence of *L. natalensis* and *L. truncatula* by sequence homology with other lymnaeid 18S accessions GenBank, Figure 3. No RFLP variation was observed for *msp*I digestion between the samples, all having a 3-banded restriction profile of fragments of 300 bp, 100 bp and 50 bp which is named as profile *msp*I type 1 [33]. In contrast, two *cfo*I restriction types were observed, Figure 3a, with *L. natalensis* having a 3-banded profile while *L. truncatula* exhibited a 2-banded profile. These restriction sites were mapped onto the sequenced region and DNA variation was located in the V2 E10-1 helix region, Figure 3b. Of note is that the *msp*I restriction site (CCGG) at position 201 is absent in these Uganda *L. natalensis* samples owing to a C to A mutation, Figure 3c. The *cfo*I restriction site (GCGC) at position 208 is intact in *L. natalensis* and combined with the *cfo*I restriction site at 161 gives rise to a 3-banded RFLP profile, previously identified as *cfoI* type 2 [33]. In *L. truncatula*, the C to G mutation leads to a loss of this *cfo*I site at position 208, hence a 2-banded RFLP profile is observed, previously recognised as *cfo*I type 1 [33]. This sequence variation is fully described in the alignment schematic of Figure 3b, with the putative secondary structure of the E10-1 helix shown in Figure 3c. Of note is the stem region of *L. natalensis* which is 3 bp longer than that of *L. truncatula* and conforms to that previously described [34,35]. No other sequence variation was found within the samples.

## Discussion

Taken together the parasitological and malacological surveys have shown that fasciolosis, resultant from *F. gigantica*, was widespread in cattle at lower altitudes in the Mount Elgon area, however, defining where the actual transmission zone ends is, however, problematic; especially so given the existence of suitable intermediate snail hosts up to 1800 m. Moreover, the confirmation of several populations of *L. truncatula* within the MENP, a single population being reported over 50 years ago [20], raises some potential concerns for transmission of *F. hepatica* should this parasite ever be introduced into this area.

**Figure 4:**
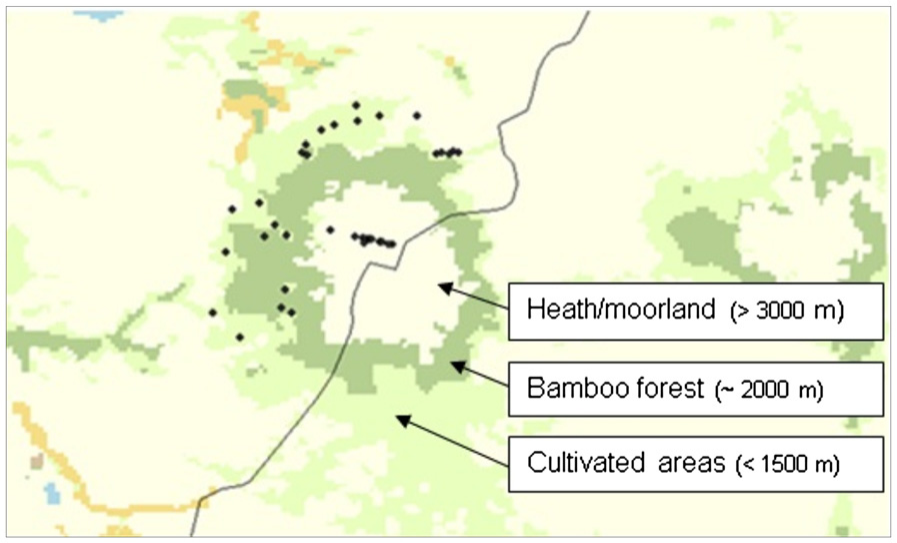
**A map derived from remotely sensed data of the changing ecozones within the study region based on GlobCover project land cover map and ground observations.** Survey sites are shown as black dots. The Uganda-Kenya border is shown.

In terms of general disease, the body condition signalment data gathered about the cattle revealed that there were a significantly greater proportion of cattle classified as ‘underweight’ at lower altitude, see Table 1. Animals at high altitude were often subjected to zero-grazing, being regularly fed on plantain leaves and were used for milk production, whereas at lower altitudes animals were free-grazing and used for meat production. Total cattle numbers in the high altitude area are estimated to be in excess of 95,000 whereas around Mbale they are thought to be slightly less in just under 65,000 animals (LM, *personal communication*). Information about the prevalence of specific signs of fluke related morbidity would be required in order to determine the extent of pathology due to fasciolosis and hence the relative importance of the parasite in the local cattle population [36,37]. Although useful for the confirmation of patently infected animals, several authors have found the sensitivity of liver inspection at the abattoir to be only 63-71% [23], thus the conditions in the two abattoirs were not conducive for ‘gold standard’ necropsy data but the available evidence can be discussed in the light of our conjoint parasitological and malacological findings, experimental serology (which might be confounded by past-infection status), and the clear ecozonation of the Mount Elgon area as shown in Figure 4 in terms of local vegetation.

### Observations associated with increasing altitude

Within the cattle sampled at altitudes between 1000 m and 1500 m, *F. gigantica* was shown to be present by all diagnostic methods used. The egg-patent prevalence by Flukefinder® was 44%, Table 2. This is in agreement with the earlier findings [18], however, between the altitudes of 1500 m and 3000 m only a single *Fasciola* egg positive cow was identified so it can be firmly considered that *F. gigantica* worms were not present in significant numbers, despite inferences from ELISA which is semi-quantitative for *F. hepatica*[29], at these elevations. This decline is concurrent with the growing scarcity of local *L. natalensis* populations, ceasing at 1800 m. Perhaps the presence of such snail hosts is a useful environmental indicator of the potential for disease transmission which can be provided relatively quickly during field-excursions as freshwater snails with dextral shell without an operculum are readily distinctive.

A key finding in this study is the dichotomy between the presence of *L. natalensis* at sites below 1500 m and *L. truncatula* at sites above 3000 m. Inspection for snails at the Sasa River Camp within MENP did not reveal the presence of lymnaeids, unlike during the 1950s where *L. truncatula* (*mweruensis*) was encountered [20]. The analysis of snail DNA very clearly confirmed the latter species likely of European origin [34,35]. The precise reasons for this lower altitudinal occurrence of *L. truncatula* are open to conjecture but could include longer-term climatic changes in the area restricting the suitable habitat to higher elevations with general climate warming. In other studies, *L. natalensis* shedding *Fasciola* have been found at altitudes of 2667 m in Kenya [38]. This perhaps indicates that factors other than altitude and the typically associated temperature are likely to be having an effect. There were wide variations in water temperature, water chemistry, vegetation (see Figure 4) and soil type.

Although altitude and temperature follow a fairly predictable trend across a wide area of East Africa, other factors such as snail habitat are much more variable, this study identified many sites where no snails were found, despite having similar temperatures to nearby sites where snails were plentiful. The apparent ‘random’ focalisation of snails in aquatic habitats is known likely to be due to cryptic micro-habitat associations and to the vagrancies of population colonisation [32]. In the regression model, for example, the input factors were a poor predictor for actual snail numbers. More generally, the presence of *F. gigantica* at altitudes above 1500 m and the absence of *F. hepatica* at altitudes above 1200 m conflicts with general model-based assumptions on temperature alone [14,22]. Micro-climates suitable for snails to survive can occur in otherwise hostile environments, but may be hard to find without more exhaustive sampling [39]. Furthermore, the ability of snails to aestivate, differences in annual transmission patterns and the timing of surveys have significant bearings upon the findings but there were no obvious differences in presence/absence of snails recorded in the March and June-July surveys.

Within the MENP and at altitudes above 3000 m, only *L. truncatula* was found alongside an enigmatic *Bulinus* population at high altitude, (see Table 4), tentatively identified of the *B. truncatus/tropicus* group [32]. Despite the presence of *L. truncatula*, there was no evidence of *Fasciola* spp. infection in the sampled buffaloes although paramphistomes were common. It could be concluded that local conditions are not suitable for the transmission of *F. hepatica* or perhaps that this parasite has yet to be introduced to this area. The MENP also extends into Kenya, where other livestock and people live together at higher altitudes. As *F. hepatica* is known to exist within natural transmission cycles in the neighbouring highlands of Kenya [40] this could be a potential route for the introduction of *F. hepatica* into the Ugandan highland region. Conversely, there was no evidence that *L. truncatula* was present at sites at lower altitudes where cattle were kept. Nonetheless, this snail species can be present at much lower altitudes in other countries [14], hence there may be some potential for this lymnaeid to establish in the 1500-2500 m zone, so further sampling should not neglect this possibility.

### Implications for control and disease surveillance

From the available evidence, no community-based intervention is currently needed for management of fasciolosis in domestic cattle at altitudes above 1500 m in this Mount Elgon area. At low altitude, however, future interventions based upon de-worming are clearly worthwhile in addition to the collection of local information on farming practices, economic impact and animal trafficking. The latter is especially important with future cattle re-stocking planned from this area to central regions of Uganda (Lira/Kitgum/Gulu) following the cessation of civil insecurities. The inspected herds at Ngenge, for example, have been ear-marked for this restocking programme.

In terms of future disease surveillance the detection of populations of *L. truncatula* in several sites frequented by UWA patrols and hiking tourists, raises some concerns of the safety of environmentally drawn water. Although the existence of *F. hepatica* has yet to be proven in this area, it would be advisable to raise awareness of fluke-borne diseases in general. More broadly, we are presently PCR screening the collected snails for evidence of fluke infections in an attempt to investigate these more cryptic aspects of parasite transmission.

## Conclusions

From parasitological sampling and observations at slaughter, infections of *F. gigantica* in cattle are common in lower altitude settings but appear to diminish with increasing elevation. This is most likely due to a growing paucity of *L. natalensis* within the environment, with a natural boundary of approximately 1800 m where no further populations of *L. natalensis* were found. Whilst *F. hepatica* was not encountered during these surveys, the presence of *L. truncatula* at elevations over 3000 m point towards a potential transmission zone within MENP should this parasite be introduced to this part of East Africa. Greater vigilance of this parasite within imported cattle, and possibly within local people, should therefore be encouraged.

## Competing interests

The authors have no competing interests.

## Authors’ contributions

The work reported here forms part of the MSc research projects of AH and JD under the joint supervision of EJLaC and JRS. Fieldwork was carried out by AH, LM, JD, EJLaC, NBK and JRS. Analysis of serological samples was assisted by JC and DJLW. Multivariate analysis was undertaken by AH with the help of LK-H, especially in spatial mapping techniques. JD, MB and JRS undertook the analysis of lymnaeid DNA analysis. All authors contributed to the production of the manuscript and revision, with JRS as final guarantor. All authors read and approved the final version of the manuscript.
